# Histone Deacetylase Inhibitors in the Treatment of Hepatocellular Carcinoma: Current Evidence and Future Opportunities

**DOI:** 10.3390/jpm11030223

**Published:** 2021-03-22

**Authors:** Nikolaos Garmpis, Christos Damaskos, Anna Garmpi, Vasiliki E. Georgakopoulou, Panagiotis Sarantis, Efstathios A. Antoniou, Michalis V. Karamouzis, Afroditi Nonni, Dimitrios Schizas, Evangelos Diamantis, Evangelos Koustas, Paraskevi Farmaki, Athanasios Syllaios, Alexandros Patsouras, Konstantinos Kontzoglou, Nikolaos Trakas, Dimitrios Dimitroulis

**Affiliations:** 1 Second Department of Propedeutic Surgery, Laiko General Hospital, Medical School, National and Kapodistrian University of Athens, 11527 Athens, Greece; efstathios.antoniou@gmail.com (E.A.A.); kckont@med.uoa.gr (K.K.); dimitroulisdimitrios@yahoo.com (D.D.); 2 N.S. Christeas Laboratory of Experimental Surgery and Surgical Research, Medical School, National and Kapodistrian University of Athens, 11527 Athens, Greece; x_damaskos@yahoo.gr; 3 Renal Transplantation Unit, Laiko General Hospital, 11527 Athens, Greece; 4 First Department of Propedeutic Internal Medicine, Laiko General Hospital, Medical School, National and Kapodistrian University of Athens, 11527 Athens, Greece; annagar@windowslive.com; 5 Department of Pulmonology, Laiko General Hospital, 11527 Athens, Greece; vaso_georgakopoulou@hotmail.com; 6 First Department of Pulmonology, Sismanogleio Hospital, 15126 Athens, Greece; 7 Molecular Oncology Unit, Department of Biological Chemistry, Medical School, National and Kapodistrian University of Athens, 11527 Athens, Greece; psarantis@med.uoa.gr (P.S.); mkaramouz@med.uoa.gr (M.V.K.); vang.koustas@gmail.com (E.K.); 8 First Department of Pathology, Medical School, National and Kapodistrian University of Athens, 11527 Athens, Greece; afnonni@med.uoa.gr; 9 First Department of Surgery, Laiko General Hospital, Medical School, National and Kapodistrian University of Athens, 11527 Athens, Greece; schizasad@gmail.com (D.S.); nh_reas@hotmail.com (A.S.); 10 Department of Endocrinology and Diabetes Center, G. Gennimatas General Hospital, 11527 Athens, Greece; vaggelisd01@gmail.com; 11 First Department of Pediatrics, Agia Sofia Children’s Hospital, Medical School, National and Kapodistrian University of Athens, 11527 Athens, Greece; evi_farmaki@hotmail.com; 12 Second Department of Internal Medicine, Tzanio General Hospital, 18536 Piraeus, Greece; patsouras.alexandros@gmail.com; 13 Department of Biochemistry, Sismanogleio Hospital, 15126 Athens, Greece; nikostrakas@sismanoglio.gr

**Keywords:** histone, deacetylase, inhibitors, HDAC, HDACI, hepatocellular carcinoma, HCC, targeted, epigenetic, anticancer, therapeutics

## Abstract

Hepatocellular carcinoma (HCC) remains a major health problem worldwide with a continuous increasing prevalence. Despite the introduction of targeted therapies like the multi-kinase inhibitor sorafenib, treatment outcomes are not encouraging. The prognosis of advanced HCC is still dismal, underlying the need for novel effective treatments. Apart from the various risk factors that predispose to the development of HCC, epigenetic factors also play a functional role in tumor genesis. Histone deacetylases (HDACs) are enzymes that remove acetyl groups from histone lysine residues of proteins, such as the core nucleosome histones, in this way not permitting DNA to loosen from the histone octamer and consequently preventing its transcription. Considering that HDAC activity is reported to be up-regulated in HCC, treatment strategies with HDAC inhibitors (HDACIs) showed some promising results. This review focuses on the use of HDACIs as novel anticancer agents and explains the mechanisms of their therapeutic effects in HCC.

## 1. Introduction

Hepatocellular carcinoma (HCC) is the predominant form of primary liver cancer, the fifth most frequently diagnosed cancer in men and the eighth most frequently diagnosed cancer in women in the United States [1]. It is also the fourth leading cause of cancer-related death in developed countries [2].

The majority of HCC cases appears in less developed areas [3]. Specifically, in men, the regions with the highest incidence are East and South-East Asia, whereas in women these regions are East Asia and West Africa [3]. Globally, the rates differ between the two genders and are more than twice as high in males [4]. According to a population-based study in the US, there is also racial and ethnic variation in the incidence of HCC. The highest HCC rate is observed among Asians/Pacific Islanders, but Hispanics and African-Americans have shown such an increase in HCC diagnosis that they are predicted to be the groups with the highest HCC rates by 2030 [5,6]. These differences are due to variation in the distribution of risk factors and in host genetics or environmental factors [7]. The main risk factors of HCC development are hepatitis B viral (HBV) infection, chronic hepatitis C viral (HCV) infection, tobacco and alcohol abuse, metabolic disorders such as diabetes mellitus, obesity and impaired glucose tolerance, uncommon genetic disorders like porphyrias, hemochromatosis, Wilson’s disease, alpha-1 antitrypsin deficiency and generally cirrhosis of almost any cause [2,8,9,10]. Considering the results from the Surveillance, Epidemiology, and End Results (SEER)-Medicare linked databases, which were used to evaluate population attributable fractions (PAF) for each risk factor, we can conclude that the largest PAF between African-Americans and Asians belongs to HCV; whereas metabolic disorders have the largest PAF between Hispanics and Caucasians [11]. Furthermore, it is also notable that the increasing prevalence of metabolic syndrome renders non-alcoholic steatohepatitis (NASH) higher in the list of the risk factors [11,12,13]. Given the fact that HCC is one of the cancers whose incidence continues to increase, as well as the high severity of this disease, alternative therapeutic targets need to be identified. The interest of the scientific community has shifted towards the mechanisms of DNA duplication and transcription in tumor genesis. It has been proven that epigenetic modifications such as acetylation and methylation of DNA affect the transcription of the genes and can be used to regulate tumor growth and its response to traditional therapeutic approaches.

Considering that treatment options for advanced HCC are extremely limited due to shortage of effective therapeutic agents and that the prognosis of advanced HCC is dismal, the need for novel therapeutic options is obvious. The role of histone deacetylase inhibitors (HDACIs) has been investigated towards this direction [14,15].

## 2. HDAC and Its Mechanism of Action

**Figure 1:**
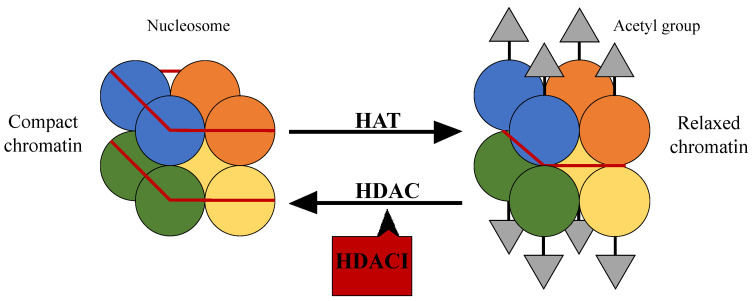
The structure of the nucleosome and the action of histone acetyltransferases and histone deacetylases.

The nucleosome and especially the control of its position, plays an important role in gene regulation. The nucleosome is the fundamental DNA structure and is wrapped around proteins called histones, which are mainly globular, apart from their N-terminal tails [16]. Histone acetylation and deacetylation through histone acetyltransferases (HATs) and histone deacetylases (HDACs) respectively, affect the DNA structure and consequently the access of transcription factors to gene promoter regions [17]. Specifically, HATs are enzymes that acetylate lysine at the ε-amino group of lysine residues leading to the neutralization of their positive charge and to a more relaxed form of chromatin. This allows the activation of the transcription of many genes, which take part in the control of cell cycle progression, differentiation, and apoptosis. HDAC on the other hand is their functional antagonist; it removes the acetyl groups, causing a more compact structure of chromatin and suppression of gene transcription [18,19,20,21] (Figure 1).

Based on their homology to yeast proteins, HDACs can be classified in 4 classes: class I consists of HDAC-1, -2, -3 and -8, class II (which is further divided into class IIa and IIb) consists of HDAC-4, -5, -6, -7, -9 and -10, class III comprises the sirtuins (SIRT-1-7) and class IV includes HDAC-11, with characteristics of both class I and II. Another classification is based on functional criteria. Classes I, II and IV of HDAC consist of the Zn^2+^-dependent classes, whereas class III differs from the other three groups of HDAC as they need a nicotinamide adenine dinucleotide (NAD+) ion for their activity [21,22,23] (Table 1).

HDAC participate in the regulation of various genes in cancer and thus they play a significant role not only as therapeutic targets, but also as diagnostic and prognostic markers. There are many human cancers that involve alteration in the expression of HDAC [20,24]. For example, an over-expression of class I HDAC has been noticed in esophageal, prostate, non-small cell lung (NSCL) and gastrointestinal cancer [25]. As far as HCC is concerned, HDAC-5 expression is increased in human HCC tissues and via up-regulation of Six1 expression, it induces high tumor proliferative capacity [26]. Furthermore, HDAC-1 and HDAC-2 could act as prognostic biomarkers, as their high expression is linked with poor survival [27,28]. Apart from this, elevated HDAC-1 expression might also be associated with poorer histological differentiation, and a more advanced TNM stage [28]. What is more, HDAC-3 has recently been proposed as a possible biomarker for recurrence following liver transplantation in HBV associated HCC [29].

## 3. HDACIs as Anti-Cancer Agents in HCC

HDACIs can affect various pathways and lead to transformed cell death. They can induce DNA damage and repair, modify gene expression, cause cell growth arrest, induce apoptosis and act as antiangiogenetic and antimetastatic factors [30]. HDACIs can be categorized according to their chemical structure into (a) hydroxamates (e.g., suberanilohydroxamic acid (SAHA)), (b) benzamides (e.g., MS275), (c) cyclic peptides (e.g., romidepsin) and (d) aliphatic acids (e.g., valproic acid (VPA)) [30]. Another way of classification is based on their specificity for HDAC subtypes or classes. Thus, SAHA and trichostatin A (TSA) are pan-HDACI, MS-275 and romidepsin inhibit class I, and VPA inhibits class I and IIa HDAC [31].

Advanced stages of clinical trials have studied several HDACIs against many cancer types, such as pancreatic [32,33], breast [34,35], thyroid [36], NSCL [37], esophageal [38], endometrial [39], colon [40] and uveal melanoma [41]. For instance, the US Food and Drug Administration has approved SAHA and romidepsin as agents against cutaneous T-cell lymphoma [42] and peripheral T-cell lymphoma respectively [43]. Considering that HDAC activity has been reported to be up-regulated in HCC, treatment strategies with HDACIs showed some promising results.

A variety of studies have demonstrated how HDACIs affect cell growth and apoptosis in HCC. Considering that the expression of HDAC-1, -2 is up-regulated in HCC, their combined inhibition was proposed by Zhou et al. According to their results, there was an increased expression of p21Waf/Cip1 and p19INK4d, which lead to decreased expression of cyclin-dependent kinases (CDKs), cell cycle blockage and apoptosis [44].

It is proved that alterations in homeostasis of glucose play an important role in the development of tumors, with the Warburg effect being one of the main causes [45]. Loss of fructose-1,6-bisphosphatase (FBP1), a rate limiting enzyme in gluconeogenesis, was found to be oncogenic in various cancer cells including gastric and colon cancer cells, suggesting that modulation of gluconeogenesis also plays an equal role in tumorigenesis [46,47]. In HCC, down-regulation of FBP1 was connected with high levels of HDAC-1 and HDAC-2. HDACIs have the ability to restore the expression of FBP1, suppress glycolysis and thus inhibit HCC growth. As a result, reduced expression of FBP1 can be a possible target in treatment of HCC with HDACIs [48].

Herein, we present the main HDACIs with anticancer effects against HCC. Table 2 summarizes their action.

### 3.1. Panobinostat

Panobinostat is a novel pan-HDACI and belongs to the cinnamic hydroxamic acid class. Panobinostat is described to have a broad anti-tumor activity against both cancer cell lines and human cancer xenografts in nude mice. Panobinostat, during a phase I study in humans with refractory hematological malignancies, demonstrated a good tolerability. The central cytotoxic effect is the acetylation of the non-histone protein Hsp90 and thus the modulation of protein chaperone function [49,50,51]. Fazio et al. tried to figure out how panobinostat influences tumor cell survival in HCC by applying it both to cancer cell lines and to xenograft models. Using human hepatoma p-53 competent HepG2 and p-53 deficient Hep3B cell lines, they noted that panobinostat inhibited proliferation via cell cycle arrest in the G1 phase. This was mediated by an increase of p21cip1/waf1, a cell cycle inhibitor. Interestingly, apoptosis was not induced through classical mitochondrial pathways, but was mediated through alternative ones. To specify this, quantitative RT-PCR indicated an up-regulation of CHOP, a marker of the unfolded protein response and endoplasmic reticulum stress, which amplified the activation of caspase-12. Moreover, dependent on the p-53 status, increased phosphorylation of H2AX and activation of the mitogen-activated protein kinase (MAPK) pathway were connected with cellular stress. When panobinostat was applied in vivo to xenograft models, there was a size decrease in treated tumor and a prolonged survival rate [52].

Over-expression of DNA methyltransferases (DNMTs), which catalyze DNA methylation on CpG islands, also plays a key role in liver tumorigenesis [53,54]. Given the fact that recent evidence indicates that HDACIs also modify non-histone proteins, the role of panobinostat in inhibition of DNMT activity in HCC cell lines was examined by Zopf et al. The results showed significant inhibition of mRNA levels for DNMT1 and DNMT3a, with a transient decrease in these protein levels as well. What is more, a decrease of the methylation status of APC was also detected. APC is one of the target genes of DNMT. The hypomethylation of APC indicated the loss of DNMT activity. Therefore, a dual mode of action of panobinostat was suggested as it interferes in epigenetic control of gene expression not only by controlling the acetylation status of histones, but also by influencing, via acetylation, the function of DNMT [55,56].

Angiogenesis has an essential role in the formation of a new vascular network and is crucial for cancer growth and metastasis. Growth factors as well as extracellular matrix components mediate this complex procedure [57]. Flt-1 and KDR, the main receptors of vascular endothelial growth factor-1 (VEGF-1), intervent VEGF signaling and are over-expressed in malignant tumors, including HCC [58]. Although different anti-angiogenic agents such as sorafenib, which targets VEGF receptors, have been used in the treatment of patients with advanced HCC, the outcomes in overall survival are limited [59]. Connecting tissue growth factor (CTGF), a member of the extracellular CCN protein family (CYR61, CTGF and NOV), also constitutes an angiogenic factor that is involved in tumor development [60]. Particularly in HCC, CTGF induces cell dedifferentiation, expression of genes participating in carcinogenesis and in vivo HCC cell growth via an autocrine loop. Epidermal growth factor receptor (EGFR) ligands promote the expression of CTGF, which is dependent on the expression of the transcriptional co-activator, Yes-associated protein (YAP) [61]. Gahr et al. investigated if panobinostat could interfere in the modulation of CTGF expression and thus inhibit angiogenesis. According to both in vitro and in vivo experiments, panobinostat induces a context-dependent differential expression of CTGF, whereas the VEGF-driven pathway plays no role in its angiogenetic activity [62].

Considering the promising effect of panobinostat in HCC, Lachemayer et al., using 3 liver cancer cell lines and a murine xenograft model, investigated its action both alone and in combination with sorafenib. The in vitro results revealed that the effect on cell viability and on induction of apoptosis, was enhanced when the combination treatment was used. However, this was not noticed on proliferation. In HCC xenografts, the treatment utilizing both agents raised the median survival, and led to a significantly lower vessel density and the highest decrease of tumor volume, in comparison to single treatments. Again, no synergism was detected as far as the proliferation index was concerned [63].

### 3.2. Trichostatin A

TSA was first discovered as an antifungal drug. Nowadays we know that it belongs to HDAC-1 inhibitors, causes cell-cycle arrest in G1 and G2 phases, and induces apoptosis via increased expression of proapoptic genes such as Bim [64,65,66].

In two studies conducted in 2004, Chiba et al. performed microarray analysis in hepatoma cell lines treated with TSA. Their results indicated altered gene expression on human hepatocytes after TSA treatment and suggested that TSA could induce cell growth inhibition and apoptosis in hepatoma cells [67,68].

Transcriptional repressors such as HDAC are gaining increasing attention as therapeutic targets in human malignancies including HCC. Given the up-regulation of HDAC-1-3 in HCC cell lines, TSA was used by Buurman et al. to inhibit them and to identify the effects of their down-regulation. This strategy led to an up-regulation of tumor suppressor miR-449. C-MET proto-oncogene, which encodes the receptor tyrosine kinase for hepatocyte growth factor, constitutes the target of miR-449 and participates in tumor development of HCC. In this study, the result of a TSA-mediated increase of miR-449 was a decrease of c-MET mRNA [66]. In another study, with the same aim of understanding the function of down-regulated HDAC-1-3, they again used TSA to increase histone acetylation. The results revealed differences in gene expression between treated and untreated cells. Reactivation of the apoptotic protease-activating factor 1 (Apaf1) showed the most significant differential expression levels. Apaf1 is the core molecule in the formation of the apoptosome, a mitochondrial caspase-activating complex. Thus, activation of Apaf-1 by TSA promoted apoptosis [69].

As well as its direct role in killing HCC cells by causing apoptosis, TSA also enhances natural killer (NK) cell-mediated killing. As the immune system plays a key role in the progression of HCC, Shin et al. focused on the immune modulating effect that TSA could have [70]. It is known that in order to avoid an immune response, many types of cancer cells down-regulate antigen-recognition associated proteins [71]. This study suggested that in TSA-treated HepG2 cells, there was an alteration in the expression of immune-associated genes. Specifically, TSA might promote the recognition of HCC cells by NK cells via up-regulation of the gene of UL-16 binding protein (ULBP) 1, which is a ligand of NK group-2 member-D cells. Furthermore, it mediates the migration of immune cells into tumor tissue in HCC. Using a xenograft model in BALB/c nude mice that lacked T cells but had functional NK cells, the authors found that TSA caused suppression of the tumor size, which was mediated by NK cells. These results clearly suggest that NK cells play a pivotal role in the anti-tumor effect of TSA in HCC both in vivo and in vitro via a TSA-mediated increase in susceptibility to NK cells [70].

The estrogen receptor a (ERa) gene has been identified as a potent tumor suppression gene in HCC and a decrease in its expression in hepatocyte cells participates in tumorigenesis [72]. The ERa gene is expressed in many tissues, particularly in the liver. One of the mechanisms of ERa silencing is hypermethylation of the CpG islands of its promoter region as well as hypoacetylation of lysine residues [72,73]. Sanaei et al. investigated whether a combination treatment of genistein, a DNA methylation agent and TSA could lead to apoptosis via reactivation of ERa expression. They used HepG2 cancer cell lines and found that, both as monotherapy and as a combination treatment, there was an increase in the expression of ERa and thus inhibition of cell growth [74]. Especially for TSA, this effect was due to over-expression of p300, which causes acetylation of ERa by blocking ubiquitination [75]. Additionally, they conducted a study examining the efficacy of curcumin compared to TSA in the reactivation of ERa. They showed that both drugs can induce apoptosis and inhibit cell growth via reactivation of ERa [76].

### 3.3. Suberanilohydroxamic Acid

SAHA is a hydroxamic acid-based hybric polar compound that belongs to the pan-HDACIs. It is able to induce apoptosis both in hematological and solid tumor cells. SAHA has shown preclinical and clinical success at tolerated doses and has received approval by the United States Food and Drug Administration for treatment of cutaneous T-cell lymphoma [77,78].

In HCC, SAHA has shown preclinical efficacy. Using three human HCC cell lines Kunnimalaiyaan et al. suggested a new possible mechanism of action of SAHA. In particular, SAHA induced apoptosis and caused decreased cellular proliferation by targeting multiple oncogenic signaling pathways. Notch signaling is one of them and according to their results, SAHA altered its signaling in both Huh-7 and Hep3B, but not on HepG2 cells. Akt kinase, a component of the PI3-K/Akt pathway had reduced activation in all cell lines. Raf-1 expression that is decreased by the action of sorafenib was also found to be reduced in Hep3B and HepG2 cell lines, making SAHA a potential additional therapeutic option in systemic treatment with sorafenib. STAT3, a member of the signal transducer and activator of transcription (STAT) protein family, is also involved in cellular growth and in regulation of apoptosis. This study demonstrated that this pathway was not inhibited by SAHA, and that it may be involved in cellular resistance and in the reduction of SAHA activity. Therefore, a combination of a SAHA and STAT inhibitor may lead to better therapeutic results in HCC [79].

SAHA is cytotoxic against HCC via the induction of NF-κB activity. Moreover NF-κB suppression can enhance the apoptosis caused by SAHA in breast cancer and heamatologic malignancies [80,81]. Sorefenib can suppress promoter induced NF-κB activity both in vitro and in vivo [82]. The aim of Hsu et al. was to investigate whether sorafenib via inhibition of this SAHA-induced pathway could increase SAHA efficacy. The results were promising as they demonstrated that sorafenib did inhibit SAHA-induced NF-κB activity via dephosphorylation of extracellular-signal-regulated kinase (ERK) both in vitro and in vivo and therefore increased its anticancer effects. Hence, these results indicate that their combination may be a new therapeutic strategy against advanced HCC [83].

The combination treatment with SAHA and sorafenib can also be supported as an option in HCC by the fact that their synergistic effect is enhanced by inhibition of autophagy. Yuan et al. examined the outcome of their combination. A raise in apoptotic rate was noticed via a decrease of anti-apoptotic Bcl-2 and increase of pro-apoptotic Bax. Additionally, there was remarkably an arrest in G0/G1 phase of the cell cycle, whereas cells in S phase and G2/M phase were decreased. Beclin-1 knockdown played a major role in the acetylation status of p53 as well as the increased expression of Bax, indicating that autophagy may participate in the additional increase in tumor cell inhibition [84].

In 2019, Freeze et al. published a study in which they examined the synergistic action of SAHA and sorafenib. They proved that SAHA reduced proliferation, migratory status and clonogenicity of tumor cells. In addition, they increased sensitivity to sorafenib. This occurred through induction of p47phox, which is a promoter of oxidative stress, and CYP2E1 over-expression [85].

Lee et al. also conducted a study concerning the efficacy of the simultaneous use of sorafenib and SAHA. The inhibition of FOXM1 rendered the tumor cells more sensitive to oxidative stress and the production of reactive oxygen species by sorafenib was accelerated in a dose-dependent way. This molecule is an important regulator of polo-like kinase 1, cyclin B1 and aurora kinase A. Furthermore, the co-administration of these drugs resulted in cell cycle arrest in phase G2/M, through the same molecular mechanism. Thus, SAHA suppressed cyclin B1 and its consequent signaling pathway, increased G2/M phase duration and decreased G1 phase duration [86]. As a result, this combination seemed to be a promising approach.

Gordon et al. also examined the possible therapeutic efficacy of the co-administration of both sorafenib and SAHA. This phase 1 study demonstrated that the co-administration of these two drugs led to increased risk of toxicity [87]. Finally, in 2020, Sanoei et al. compared the anti-oncogenc effect of 5-aza-2 deoxycytidine and SAHA in hepatocellular malignant lines. They proved that both drugs exert an apoptotic effect and inhibit cell viability through many pathways such as down-regulation of DNMT1, DNMT3a, and DNMT3b [88].

### 3.4. Valproic Acid

VPA is a commonly known antiepileptic drug as well as a mood-stabilizing drug [89]. It is now also considered a class I and class II HDACI and its use has been investigated in various cancers such as cervical cancers and small cell lung carcinomas [90,91]. Despite the fact that VPA is identified as a hepatotoxic drug [92], it is demonstrated to lead to apoptosis and growth inhibition in HCC cells [93,94]. Notch signaling is a pathway that takes part in pathogenesis of HCC and up-regulation of Notch3 and Notch4 mRNA has been detected in HCC [95,96,97]. However, there are studies that suggest Notch signaling is a tumor-suppressor [98,99]. Despite its controversial functions, Sun et al., using HCC HTB-52 cells with a high Notch signature, examined whether Notch signaling was involved in VPA-mediated cell growth repression. The results showed that VPA down-regulated the expression of Notch1 and Notch2 as well as the Notch target gene HES1. The effect of Notch-induced cell proliferation was also reversed even when they added ICN1, the over-expressing Notch1 active form, in treatment with VPA. Apart from that, during the study they found that VPA induced the expression of G protein-coupled somatostatin receptor 2 (SSTR2). Therefore, a receptor-target systematic therapy with VPA and SSTR2 may be used, taking advantage of the effect of VPA as receptor up-regulator [100]. Yang et al. also proved that VPA down-regulates the NOTCH/AKt signaling pathway. They also showed that co-administration with sorafenib can have an additional effect against malignant hepatocellular cells [101]. Liu-Jing et al. also examined the possible mechanisms of action and therapeutic results of the simultaneous use of sorafenib and VPA. They demonstrated that this combination down-regulated the Jagged2-mediated Notch1, which caused suppression of tumor growth [102].

Whether VPA can amplify the radiation effect was also studied. The synergistic effect of VPA with two types of radiotherapy, photon and proton, in HCC was evaluated by Yu et al. using both in vitro and in vivo models. The strongest radiosensitization was when it was combined with proton radiation rather than photon radiation. The rise in DNA damage, caused by accumulation of reactive oxygen species (ROS), was evaluated by the increase in formation of γ-H2AX foci, a biomarker of DNA double strand breaks. The involved mechanism was down-regulation of NRF2 by VPA, suggesting that NRF2 may protect cells from proton-induced killing [103].

Another combination that was proposed in treatment of HCC was the addition of VPA in doxorubicin. In a study conducted by Saha et al., they attempted to clarify the mechanism of their synergistic action. According to the results, their synergistic cytotoxicity was due to increased ROS generation, up-regulation of autophagy pathway and induction of the caveolae-mediated endocytosis pathway [104]. Finally, a combination of VPA hydralazine in combination with gemcitabine and cisplatin followed by doxorubicin and dacarbazine for advanced hepatocellular carcinoma was tested [105]. The results were promising since the overall survival was prolonged, without an increase in the adverse effects.

### 3.5. Resminostat

Resminostat is a novel oral HDACI, inhibiting classes I, IIa and IV of HDAC. Its antitumor efficiency was first demonstrated in multiple myeloma [106]. The mechanism of its effect in HCC was studied by Fu et al. in three cancer cell lines. Its cytotoxic action was restricted only in cancer cell lines due to the low expression rate of HDAC-1, -2 and -3 in non-cancerous human hepatocytes. The promotion of apoptosis was noticed by the rise in the activity of caspase-3 and -9 and by the release of mitochondrial cytochrome-C. Furthermore, the mitochondrial transition pore (mPTP)-dependent apoptosis pathway was also activated by resminostat. This was confirmed with the use of cyclosporine-A and sanglifehrin-A, which are mPTP blockers. After their use, the resminostat-associated induction of apoptosis was inhibited [107].

Resminostat is under phase I/II clinical study (SHELTER study). Given the fact that sorafenib, which is used in patients with advanced HCC, provides an overall survival of 10.7 months and that many of these patients have resistance to its action [108], the need for identifying alternative therapeutic options is high. Towards this direction, Bitzer et al. investigated the safety and pharmacokinetics of resminostat as well as the efficacy of its combination with sorafenib in a trial with 57 patients with advanced HCC. All of them had either unresectable locally advanced or metastatic HCC and they were diagnosed histologically or clinically following AASLD criteria. They also had radiologically confirmed tumor progression on first line treatment with sorafenib. According to the results, resminostat both as single therapy and as combination treatment was safe and well-tolerated at all dose levels studied. Furthermore, the combination treatment led to a disease control rate of close to 87.5% in these patients. Finally, ZFP64 was detected to be a highly regulated gene by resminostat. In particular, high baseline levels were combined with longer overall survival. This suggests that ZFP64 may act as a biomarker for the response to resminostat. Nevertheless, its use needs to be further investigated [109].

Soukupova et al. tried to identify the molecular mechanism behind the synergism of resminostat and sorafenib that the SHELTER study had showed. Considering that resistance to sorafenib was previously associated with mesenchymal phenotype and expression of stem related gene CD44 [110], their study focused on resminostat-induced regulation of epithelial-mesenchymal and stemness phenotype as a way to sensitize sorafenib. The results revealed that treatment with resminostat in mesenchymal HLE and HLF cells caused a shift from mesenchymal to epithelial phenotype. In detail, in HLE cells, up-regulation of CDH1 and down-regulation of TWIST1 and SNAI2, epithelial to mesenchymal transition (EMT)-inducing transcription factors, was detected. In HLF cells resminostat treatment led to up-regulation of CDH1 and down-regulation of VIM. There was also increased E-cadherin and decreased vimentin expression. What is more, resminostat down-regulated CD44 expression. Through these processes, resminostat makes mesenchymal HCC more sensitive to sorafenib-induced apoptosis [111].

### 3.6. Other HDACIs

AR-42 is an orally available phenylbutyrate-derived HDACI [112]. Lu et al. demonstrated its synergistic action with radiation in inhibition of HCC cell growth. In detail, they used both Huh7 cancer cell lines and xenograft models. The pre-treatment with AR-42 had a radiosensitizing effect. Their in vitro combination led to activation of caspase-3 and cleaved poly (ADP-ribose) polymerase (PARP) and, following this pathway, the promotion of apoptosis. Moreover, the combination treatment increased DNA damage and suppressed regrowth of HCC cells. The use of AR-42 also increased cell death caused by radiation via inhibition of DNA end-binding activity of a regulatory protein for DNA repair, Ku70. As far as the in vivo experiment is concerned, further inhibition of the growth of the tumor was noticed when the combination treatment was used, and this result was due to hyperacetylation of Ku70 [113].

Droxinostat is a selective HDACI that inhibits HDAC-3, -6 and -8 [114]. It sensitizes PPC-1 cells to FAS and TRAIL and also has effects in other cell lines including T47D, DU-145 and OVCAR-3 [115,116]. Liu et al. probed the efficacy of droxinostat in HCC cancer cell lines. They indicated that levels of HDAC-3 were suppressed and that acetylation of histones H3 and H4 was promoted. The involved mechanism in the induction of apoptosis was shown to be the activation of the mitochondrial apoptotic pathway and the down-regulation of FLIP expression. In detail, the role of the mitochondrial p53 pathway was suggested by the up-regulation of phosphor-p53 and cleaved caspase 3 protein and the down-regulation of Bcl-2 [117].

Romidepsin is an HDACI that has received approval in refractory cutaneous and peripheral T cell lymphoma [118]. Its role in HCC has been investigated by Sun et al. and thus they rendered it as a potential option in the systematic treatment of HCC. In detail, they proved its activity both in vitro and in vivo. The identified mechanism of action was the induction of cell cycle arrest in the G2/M phase via modifications in p21/cdc25C/cdc2/cyclin B proteins as well as the promotion of apoptosis through the JNK/c-Jun/caspase 3 pathway. All of these results were time and dose dependent. There was down-regulation of PI3K, NF-κB p65, c-Jun, c-met and BAD apoptotic molecules and up-regulation of PTEN and CYLD [119]. The in vivo results showed a decrease in the tumor size in mice treated with romidepsin and no related toxicities [120].

Belinostat is a pan-HDACI, which has been tested in various solid and hematological malignancies [121]. Phase II/III clinical trials of belinostat against HCC have been conducted without encouraging results [122]. Llopiz et al. examined the synergistic effect of belinostat with checkpoint inhibitors. Belinostat increased the efficacy of anti-CTLA-4 agents and enhanced antitumor immunity through down-regulation of T-regulatory cells and an increase in interferon gamma (IFN-γ) production. Moreover, an increased infiltration of antigen-presenting cells and tumor-infiltrating cells, including macrophages, was noticed, rendering its simultaneous use with immune checkpoint inhibitors an effective therapeutic approach. Specifically, their research proved that their combined use caused complete tumor rejection [123].

Quisinostat is an HDACI that demonstrated promising results. In 2018, He et al. proved that quisinostat alone or combined with sorafenib could be effective against HCC. Specifically, quisinostat altered the pathways of PI3K/AKT/p21and JNK/c- jun/caspase3, leading to G0/G1 cycle arrest and apoptosis. Bcl2 and Bcl-xl were reduced, whereas Caspase-3 and -9 were over-expressed. Additionally, a synergistic activity with sorafenib was noticed in both in vivo and in vitro experiments. The anti-proliferative effect was a result of increased expression of JNK phosphorylation, whereas the anti-apoptotic one was due to facilitation of the JNk pathway [124].

## 4. Conclusions

The high incidence and lack of effective chemotherapeutic agents in advanced HCC has led to interest in the field of HDACIs. The first results are promising. The use of biomarkers would be a method to identify the optimal clinical application of HDACIs. Novel designed trials need to be conducted and patients who are most likely to benefit from them need to be identified. Thus, HDACI will be established as a targeted therapeutic approach against HCC, with maximum anti-cancer activity and minimum side-effects.

## Figures and Tables

**Table 1:** Classification of histone deacetylases.

Class					Dependence
I	II		III	IV	
	A	B			
HDAC-1 HDAC-2 HDAC-3 HDAC-8	HDAC-4 HDAC-5 HDAC-7 HDAC-9	HDAC-6 HDAC-10		HDAC-11	Zn^2+^
			SIRT-1 SIRT-2 SIRT-3 SIRT-4 SIRT-5 SIRT-6 SIRT-7		NAD+

**Table 2:** Main histone deacetylase inhibitors with anticancer effects against hepatocellular carcinoma.

HDACI	Anti-HCC Effect
Panobinostat	Cell proliferation inhibition Cell cycle arrest Apoptosis
Trichostatin A (TSA)	Cell growth inhibition Apoptosis
Suberanilohydroxamic acid (SAHA)	Cell proliferation inhibition Cell cycle arrest Apoptosis
Valproic acid (VPA)	Cell growth inhibition Apoptosis
Resminostat	Apoptosis
AR-42	Cell growth inhibition Apoptosis
Droxinostat	Apoptosis
Romidepsin	Cell cycle arrest Apoptosis
Belinostat	Antitumor immunity
Quisinostat	Cell cycle arrest Apoptosis
